# The effect of a short-wave filtering contact lens on color appearance

**DOI:** 10.1167/jov.23.1.2

**Published:** 2023-01-03

**Authors:** Billy R. Hammond, John Buch, Lisa M. Renzi-Hammond, Jenny M. Bosten, Derek Nankivil

**Affiliations:** 1 Vision Sciences Laboratory, Behavioral and Brain Sciences Program, Department of Psychology, University of Georgia, Athens, GA, USA; 2 Research & Development, Johnson & Johnson Vision Care, Inc., Jacksonville, FL, USA; 3 Institute of Gerontology, Department of Health Promotion and Behavior, University of Georgia, Athens, GA, USA; 4 University of Sussex, Falmer, UK; 5 Research & Development, Johnson & Johnson Vision Care, Inc., Jacksonville, FL, USA

**Keywords:** color vision, color appearance, blue-light filter, short-wave filter, contact lenses

## Abstract

We assessed the effect of a contact lens that filters short-wavelength (SW) visible light on color appearance. These effects were modeled and measured by direct comparison to a clear contact lens. Sixty-one subjects were enrolled, and 58 completed as cohort; 31 were 18 to 39 years old (mean ± SD, 29.6 ± 5.6), 27 were 40 to 65 years old (50.1 ± 8.1). A double-masked contralateral design was used; participants randomly wore a SW-filtering contact lens on one eye and a clear control lens on the other eye. Subjects then mixed three primaries (including a short-wave primary, strongly within the absorbance of the test lens) until a perceived perfect neutral white was achieved with each eye. Color appearance was quantified using chromaticity coordinates measured with a spectral radiometer within a custom-built tricolorimeter. Color vision in natural scenes was simulated using hyperspectral images and cone fundamentals based on a standard observer. Overall, the chromaticity coordinates of matches that were set using the SW-filtering contact lens (*n* = 58; *x* = 0.345, *y* = 0.325, *u*′ = 0.222, *v*′ = 0.470) and clear contact lens (*n* = 58; *x* = 0.344, *y* = 0.325, *u*′ = 0.223, *v*′ = 0.471) were not significantly different, regardless of age group. Simulations indicated that, for natural scenes, the SW-filtering contact lens that was evaluated changes L/(L+M) and S/(L+M) chromatic contrast by no more than −1.4% to +1.1% and −36.9% to +5.0%, respectively. Tricolorimetry was used to measure color appearance in subjects wearing a SW-filtering lens in one eye and a clear lens in the other, and the results indicate that imparting a subtle tint to a contact lens, as in the SW-filtering lens that was evaluated, does not alter color appearance for younger or older subjects. A model of color vision predicted little effect of the lens on chromatic contrast for natural scenes.

## Introduction

Tinted filters are often used in photography to alter the color appearance of images. It has also been hypothesized (e.g., Simunovic et al., 2012) that intraocular filters, such as blue-light filtering intraocular lenses (BLF IOLs) or lenticular or retinal macular pigments (MPs) have the potential to alter color perception. Bornstein (1973) argued, for example, that semantic differences in basic color terminology across cultures might be explained by group differences in average MP levels. Bornstein hypothesized that the high MP levels of some groups (e.g., individuals near the tropical equator who have a high intake of carotenoid-rich foods) would cause “a reduction in the perception of blueness.” The increasing yellowness of Claude Monet's lens has often been used as an explanation for the slow disappearance of shorter wave colors in his impressionistic paintings (Steele & O'Leary, 2001). The retinal and lenticular pigments do, in fact, screen the foveal cones from a significant amount of light in the short-wave (SW) end of the visible spectrum (400–500 nm). Further, this amount can dramatically differ between individuals (Curran Celentano, Burke, & Hammond, 2002). At peak absorbance (460 nm, the dominant wavelength of blue sky light), MP screening (Hammond, Wooten, & Snodderly, 1997) may vary by more than 1 log unit of optical density (= 0.2 to over 1.3). When one adds SW screening of visible light by the crystalline lens (= 0.9 to over 1.6 at 407) (Wooten, Hammond, & Renzi, 2007), this creates a situation of fairly dramatic individual differences in the amount of SW light incident on the photoreceptors. Note that, although the eye therefore naturally filters light at 460 nm, the same wavelength associated with peak melatonin suppression (Rea, Bullough, & Figueiro, 2002), the test lens in this study does not. Nonetheless, empirical data show that these natural individual differences seem to have very little impact on color perception. For example, Stringham, Hammond, Wooten, and Snodderly, (2006) and Stringham and Hammond (2007) measured π^1^ sensitivity and hue cancellation values in subjects with a wide range of MP density. Comparisons were made both across and within subjects (different locations on the retina, where MPs were either dense or mostly absent, were compared). No relation was found; the visual system presumably increases gain to offset filtering by even the highest levels of MP density. The visual system does not operate like a passive detector; rather, it can adapt to large variations in ambient lighting and compensate for similarly large variations in stable intraocular filters.

The light-filtering characteristics of the natural lens, tinted intraocular implants, and macular pigment are all relatively stable features of the eye. When filtering changes quickly, however, the system requires time to renormalize. Delahunt, Webster, Ma, and Werner (2004) found that chromatic mechanisms following cataract surgery and implantation of a clear IOL took months to reset. Tregillus, Werner, and Webster (2016) used yellow spectacle lenses and tested renormalization each day over the span of a week. They found that some adaptive processes (such as simultaneous contrast) were quite fast (minutes), but full adaptation to the lenses (color perception similar to no lenses) often took hours (this process may be accelerated in individuals who habitually wear tinted lenses) (Engel, Wilkins, Mand, Helwig, & Allen, 2016). The results of Tregillus et al. (2016) indicate that alterations in color perception might be different with lenses that are extrinsic to the eye, such as spectacles or contact lenses, compared to intraocular filters that are stable over time. Alzahrani et al. (2020) modeled the effects of commercially available BLF lenses on the perception of color and concluded that they are “capable of reducing the perception of blue colours … by 5-36 per cent.” Thus, the literature may lead the practitioner to ask whether such filters affect color perception.

**Figure 1.**
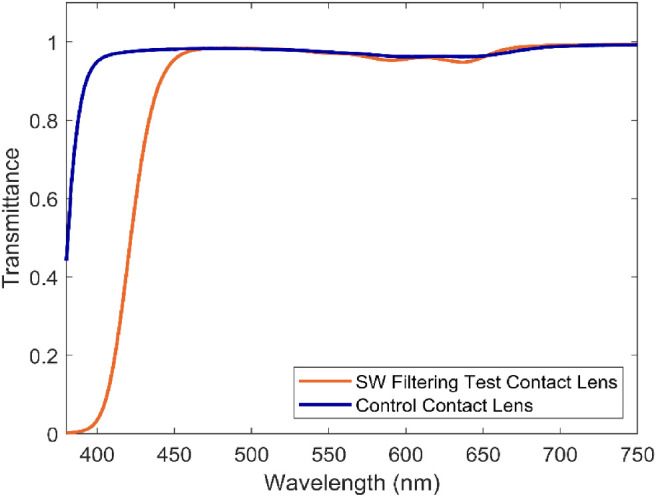
Transmittance of the SW-filtering (orange) and control (blue) contact lenses.

SW-filtering contact lenses contains chromophores that intentionally absorb specific wavelengths (380–450 nm) of visible light. Chromophores that filter in this range have a yellowish hue or “tint” imparted to them when viewed against a white background. The test lens also contains a bluish compound to normalize the cosmetic acceptance of the lens, thus resulting in a slightly teal color. This should not be confused with “tinted” contact lenses that contain pigments or dyes for the purpose of changing cosmetic appearance or making a lens easier to see for handling. See Figure 1 for the spectral transmission of the test and control lens used in this study. The test lens has the highest filtering (∼60%) within the range of 380 to 450 nm of any known soft contact lens. The control lens contains an ultraviolet-absorbing compound and a blue visibility tint and is considered relatively clear compared to the test lens.

In this study, we assessed whether SW-filtering contact lenses would influence color appearance (as opposed to chromatic discrimination measured by tests such as the FM-100 hue test). Two approaches were used. The first was to measure color appearance using additive tricolorimetry where three color primaries (red, green, and blue) are mixed to achieve a perfect neutral white. Subject settings are plotted in CIE space as chromaticity coordinates. This type of geometric representation of color space allows specification of the appearance of any mixture of light, rather than its spectral composition, by specific coordinates. Disruptions from normal trichromatic color vision are represented as alterations in the relative amounts of the color primaries used to create a perfect perceived neutral white. Because each variable is on a continuous scale, even small alterations in color appearance can be assessed.

Although it is informative to characterize the effectiveness of such filters using precisely controlled and calibrated stimuli, any functional impact of tinted lenses will be realized in the natural world through their effect on light in natural scenes. To estimate the impact of a filter on color perception in natural environments, we also modeled the visual responses of individual observers to light in the real world. Thus, as a complement to the aforementioned empirical study, we developed modeling tools that permit quantitative characterization of the effect of filters on the color vision of individually specified observers. We present summary statistics of the predicted effects of the tested SW-filtering lenses on the color vision of a standard observer for a series of hyperspectral images of natural scenes.

## Methods

### Design of the empirical study and subjects

A prospective, randomized, double-masked, contralateral design was used. Subjects were habitual wearers of spherical silicone hydrogel soft contact lenses with best-corrected visual acuity of 20/25 or better in each eye. Ishihara pseudoisochromatic plates were used to screen for color vision deficiency, and a basic clinical exam was used to exclude any overt ocular pathology (no subjects had to be excluded). A total of 61 subjects were enrolled from one clinical site (Georgia Center for Sight, Greensboro, GA). All enrolled subjects were randomly assigned to one of the two lens sequences (test/control or control/test) and completed the study (the lenses were worn throughout the single test session). All 61 subjects were included in the safety population, which included 31 subjects 18 to 39 years old (50.8%) and 30 subjects 40 to 65 years old (49.2%). Two age groups were selected to assess any change due to age; for example, lens yellowing with age is a possible additive influence (Artigas, Felipe, Navea, Fandino, & Artigas, 2012). Of the 61 subjects, three subjects were excluded from the per-protocol population due to lens dispensing with incorrect product codes and missing data. The per-protocol population (cohort) included 58 subjects, with 31 subjects 18 to 39 years old (29.6 ± 5.6; 53.4%) and 27 subjects 40 to 65 years old (50.1 ± 8.1; 46.6%). Of the 58 subjects from the per-protocol population, 44 were females (75.9%), and 14 were males (24.1%). The majority of subjects were white (42; 72.4%) and non-Hispanic or Latino (56; 96.6%), and their average age was 39.2 ± 12.34 years. The available contact lens powers were −1.00 through −6.00 diopters (D) in 0.25-D steps and were fit to achieve a plano spherical over-refraction OD and OS. There were no adverse events.

### Ethics

The study was performed in accordance with “Clinical investigation of medical devices for human subjects” (ISO 14155:2011) and followed the tenets of the Declaration of Helsinki. Written informed (and verbal) consent was obtained from all subjects. The protocol was approved by the Sterling Institutional Review Board, Atlanta, GA.

### Apparatus and procedure

A custom designed tricolorimeter was constructed to determine and specify the locus of perceptual white within the CIE chromaticity diagram (for additional details and a schematic, see Hammond, Wooten, Saint, & Renzi-Hammond, 2021). The optical system was built around two integrating spheres (Labsphere, North Sutton, NH). Each hemisphere was drilled for two apertures (1-inch diameter). The light source was a 1-inch-diameter, chip-on-board array of cool white light-emitting diodes (LEDs; 6500 color temperature). This light array was reflected within the sphere and diffused in all directions creating a Lambertian emitter where the luminance toward an observer was independent of the viewing position; the perception was one devoid of all texture and perfectly uniform. This light passed through a filter assembly composed of a red (R), a green (G), and a blue (B) filter (Wratten filters; #26, #40, and #47, respectively; Edmund Optics, Barrington, NJ). The tripartite filter assembly was mounted onto a vertical/horizontal micrometer. Adjustment of the filter along the horizontal direction resulted in either (1) an increase in red and a decrease in green, or (2) a decrease in red and an increase in green. Adjustment of the filter along the vertical direction resulted in either (1) an increase in both red and green and a decrease in blue, or (2) a decrease in both red and green and an increase in blue. With this, a wide ratio of the three colored filters could be set to sample a large subset of the CIE chromaticity diagram ranging from clearly red or green or blue to a perfect white and all colors in between. Optical baffles were used to prevent stray light between the various components. After passing through the RGB filter, light entered the second sphere, which served to additively mix the R, G, and B components thoroughly so that the emitted light was also Lambertian and color appearance was constant across the perceived target for a given RGB setting.

Light from the second sphere was transmitted through a lens and then passed into a beam splitter where half the light was reflected onto a second lens that focused the light on the detector of a spectral radiometer, which calculated the chromaticity coordinates. The other half of the collimated light was directed through an eye cup and into the eye. For an emmetrope, the image would be in sharp focus. For a myope or a hyperope, however, the image would be in front of the retina or behind the retina, respectively. This design allowed for control over the focus of the image for any observer by simply increasing or decreasing the distance between the aperture of the second sphere and the final lens. It is one example of a class of telecentric lens assemblies, and it provided an image magnification that was constant in size no matter the distance between the aperture and lens. For our application, the eye cup was fixed because it was the reference point for eye position. Therefore, we mounted the spheres on a platform that could be translated along the *z*-axis. The observer varied the lens position by turning a dial until perfect focus was achieved.

### Overview of the psychophysical technique

Subjects wore the contact lenses for approximately 25 minutes before beginning an experimental session with the tristimulus colorimeter. Before the start of the session, the experimenter demonstrated to the subject how turning the knob changed the color appearance of the test stimulus and explained that the goal was to identify “pure white” (or “snow white,” no identifiable tints or colors). The tester then moved the control knobs to achieve a maximal saturation starting point that participants reliably identified as “red.” From that point, the subject verbally guided the experimenter's adjustments until no hint of hue was perceived, with a criterion point of “pure white.” The same procedure (below) was repeated for maximal saturation starting points that participants reliably identified as red, green, and blue.

### Finding “white”

From each of these three starting points, the experimenter systematically adjusted one axis (e.g., red–green) at a time and instructed the subject to state when the visual field was as close to white as possible along that axis, at which point the experimenter stopped turning that dial. Then, the experimenter moved to the other axis (red–green–blue in this example) and adjusted that dial until the visual field was as close to white as possible. This continued, back and forth, with small, fine-tuned adjustments, until the subject reported that the visual field was pure white without any tint of color.

When an approximate white setting was obtained, subjects were asked to look away from the device for approximately 5 seconds, and then back into the eye piece to double check that the visual field did not contain any tint of color at that setting. They were asked such questions as, “If you had to give what you are seeing a color name, what would you call it?” Also, using that setting, the experimenter turned each knob to bracket the area (meaning, at the point where the subject first perceived a tint). Four trials, one from each starting point, were collected for each eye. For each measurement, the spectroradiometer provided measures of the stimulus color as in chromaticity coordinates (*x*, *y* and *u*′, *v*′) and stimulus illuminance (lux).

### Design of the modeling study: Simulating color vision in natural scenes

#### Hyperspectral images

Any shifts in color chromaticity conferred by the SW-filtering lens may not equate to changes in color appearance, as chromatic adaptation may rebalance the color appearance to compensate for initial changes in receptor signals caused by the filter. However, the colored filter may still affect the range of color contrasts available to the observer. We were interested in predicting the effect of the filter on the available color gamut for natural scenes. Colors in RGB images have very different spectra from those in natural scenes, and correspondingly the effects of filters on RGB images will be very different from their effects on natural scenes. It is therefore not possible to fully simulate the effect of filters on the color perception of natural scenes solely from RGB images; instead, hyperspectral images are required, where full spectral information is available at each pixel. To this end, we used five sets of hyperspectral images. Three images sets were obtained from the literature (Chakrabarti & Zickler, 2011; Nascimento, Ferreira, & Foster, 2002; Parraga, Brelstaff, Troscianko, & Moorhead, 1998), and one image set was acquired in support of this study. The image sets obtained from the literature were chosen from those available for their high precision and lack of spectral artifacts.

A calibrated IQ camera (Specim Spectral Imaging Oy, Ltd., Oulu, Finland) was used to gather a custom set of hyperspectral images of scenes created in the lab under controlled lighting and outdoor scenes. The custom image set consisted of 35 images featuring a mixture of manmade and colored objects, including objects where color discrimination is often critical (e.g., maps, colored markers, flowers, traffic lights, colored threads and cables, fruit). The spatial resolution of the images was 512 × 512, and the native spectral resolution was 204 wavebands between 400 and 1000 nm.

**Figure 2.**
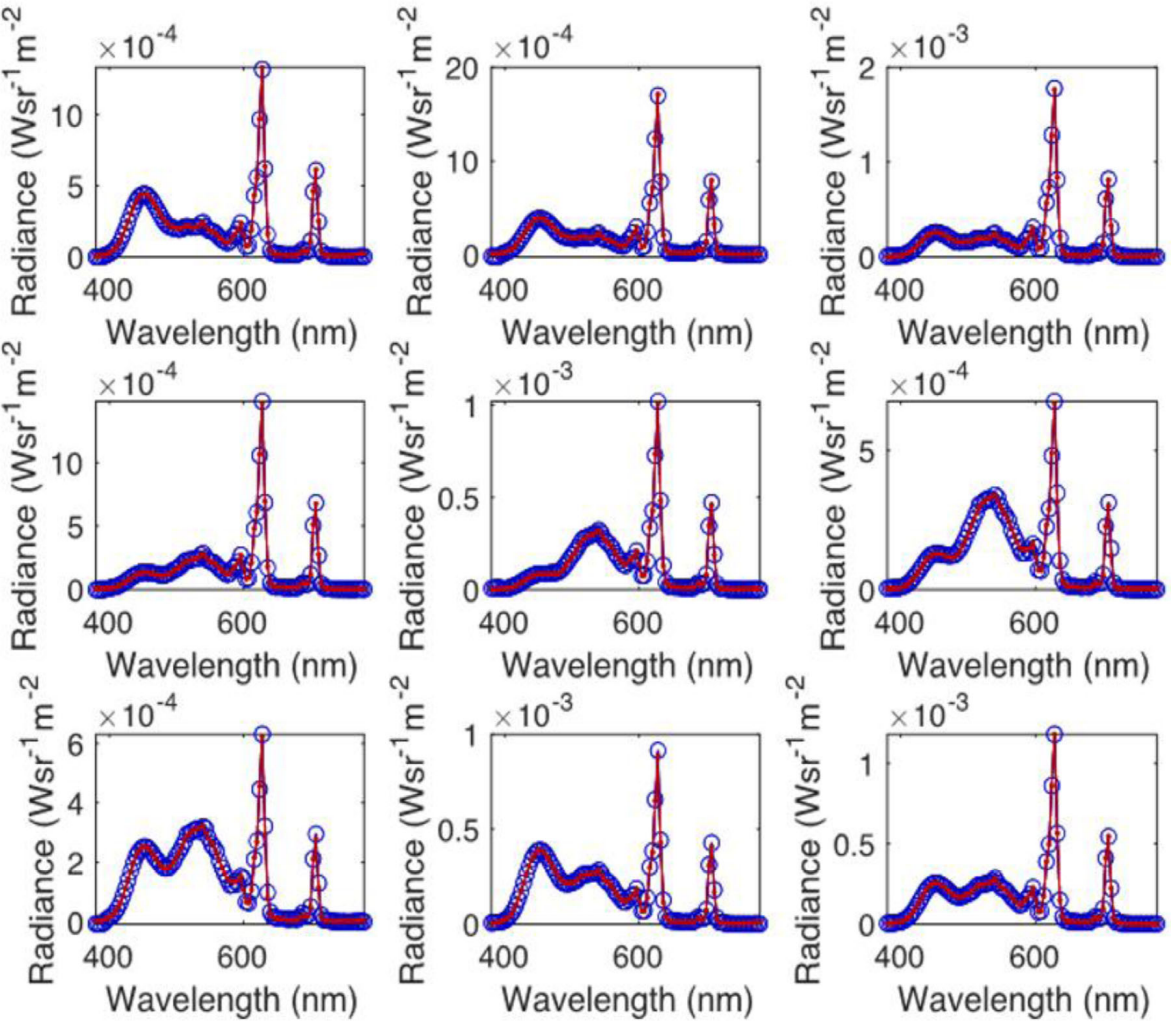
Calibration of Specim IQ. Nine colored stimuli were presented on a Mitsubishi Diamond Pro CRT monitor. The spectra of the stimuli are shown measured using a PR655 spectroradiometer (red line) and using the calibrated Specim IQ hyperspectral camera (blue open circles).

These custom images were calibrated using a PR655 spectroradiometer (PhotoResearch, Chatsworth, CA), and colored stimuli were presented on a cathode-ray tube (CRT) monitor (Mitsubishi, Tokyo, Japan). By measuring a series of the same stimuli with both the PR655 and the Specim IQ, a vector of mean wavelength-specific scaling factors was calculated that allowed the camera-specific intensities of the Specim IQ to be transformed into radiance units. The wavelengths output by the Specim IQ were not calibrated, but the calibration was checked using an independent set of stimuli (Figure 2).

The two sets of publicly available hyperspectral images captured by Foster, Amano, Nascimento, and Foster (2006) are mostly of outdoor scenes including foliage, flowers, countrysides, and cityscapes. The images are 820 × 820 spatially with 33 spectral wavebands between 400 and 720 nm. The Nascimento et al. (2002) dataset consists of eight images with reflectance information only (i.e., as if the scenes were imaged under equal energy white). The Parraga et al. (1998) dataset consists of 29 images of outdoor scenes, including trees, foliage, flowers, bark, and soil. The images provided radiance for 31 wavebands between 400 and 700 nm with a spatial resolution of 256 × 256. The Chakrabarti and Zickler (2011) dataset includes 25 indoor images and 38 outdoor images. Several images were eliminated from Chakrabarti and Zickler's full set because they contained spectral artifacts (camera saturation). The spatial resolution of the images was 1040 × 1392 with intensities at 31 wavebands between 420 nm and 720 nm. The images were calibrated to account for camera sensitivity but were not radiance calibrated.

#### Individual observer

**Figure 3.**
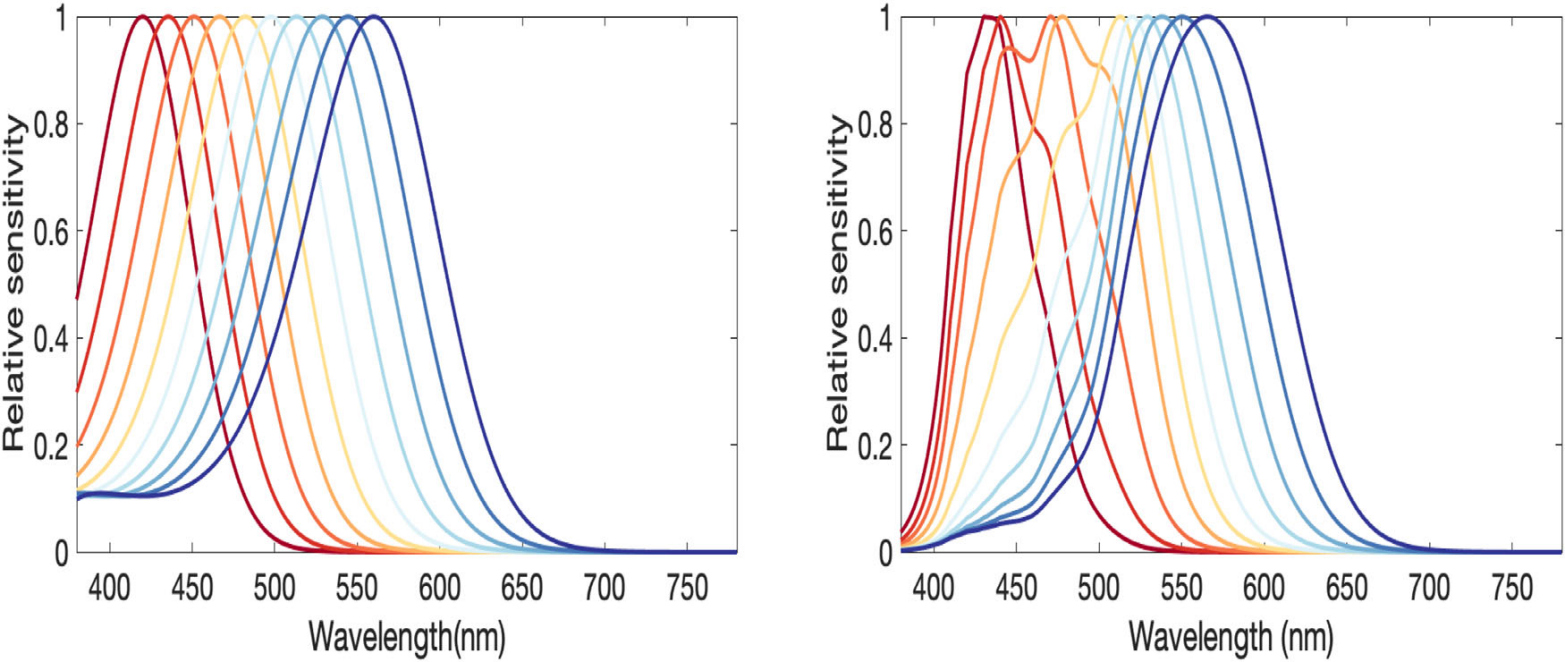
(Left) Stockman and Sharpe (2000) nomogram for simulating cone fundamentals of arbitrary peak sensitivity. (Right) The same simulated cone fundamentals for a 20-year-old observer with a macular pigment density of 0.35 and optical density of 0.38.

The individual observer model was based on the Stockman and Sharpe (2000) nomogram (Figure 3). The standard normal observer was defined using in vitro peak receptoral sensitivities of 558.9 nm, 530.3 nm, and 430.7 nm. Results of the nomogram, showing example normalized cone fundamentals created between 420 nm and 560 nm, are shown in Figure 3. Additional model features included macular pigment optical density (= 0.35); age-dependent lens density function using equations provided by Pokorny, Smith, and Lutze (1987) (e.g., 20 years); receptoral optical density (e.g., 0.38, 0.38, and 0.3 for L, M, and S as recommended by Stockman and Sharpe, 2000); and scaling factors for the relative peak heights of the L and M cone fundamentals so that L = 1.5M as recommended by Stockman and Sharpe (2000). The scaling factor for the S-cone fundamental is arbitrary.

#### Quantifying the impact of a filter on color vision

After defining the observer, each hyperspectral image was transformed to observer-specific LMS values (with and without the additional filter). First, the observer-specific matrices of MacLeod and Boynton (1979) chromaticity coordinates were created and translated to center the white point at (0, 0), which was calculated as the average MacLeod–Boynton chromaticity coordinates S/(L+M) and L/(L+M) over all pixels in the image. Gamut changes were then calculated relative to the white point. The impact of the filter was calculated as the difference between the absolute MacLeod–Boynton chromaticity coordinates of the scene with the filter and the absolute MacLeod–Boynton chromaticity coordinates of the scene without the filter. Using the absolute values ensures that any enhancement in chromatic contrast from white (along the bipolar chromaticity dimension) is a positive number in the difference metric, whereas any reduction in chromatic contrast from white is a negative number. The size of the change in pixel chromatic contrast caused by the filter was quantified as a percentage of 95% of the gamut of the scene without the filter, which was calculated as difference between the 2.5th and 97.5th percentiles of the MacLeod–Boynton chromaticity coordinates of all pixels in the image. Simulations were conducted for macular pigment optical density = 0.35 and lens age = 20.

## Results

### Empirical study

**Figure 4.**
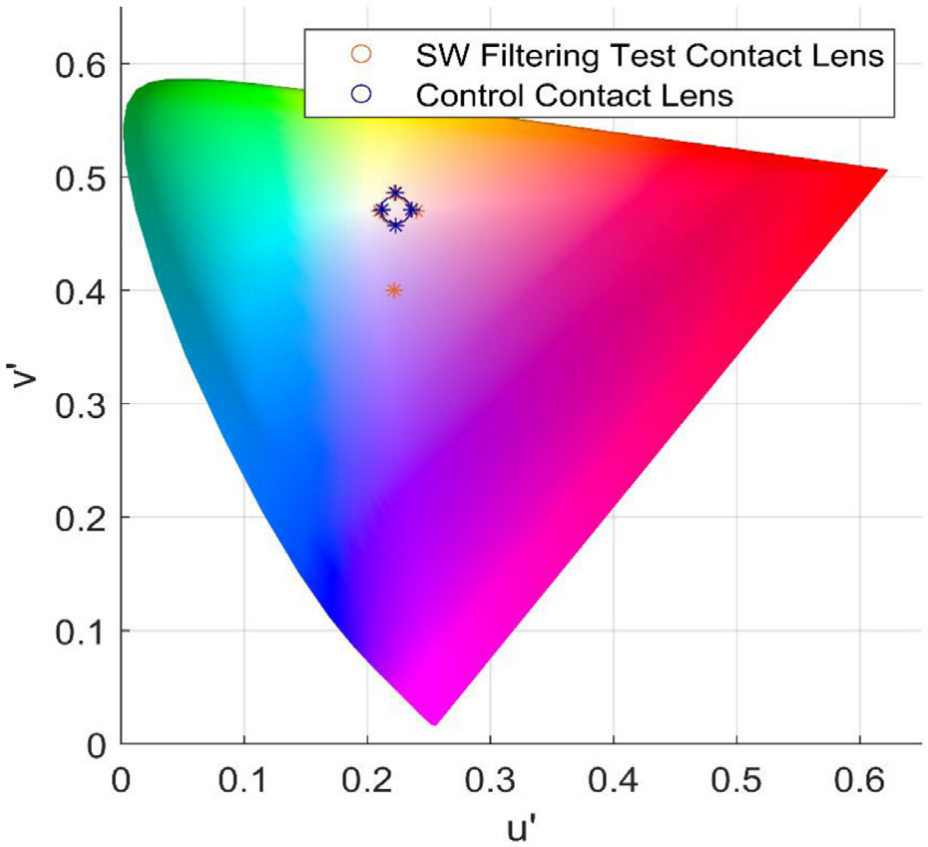
Subjective white point of all subjects. The size of the marker (open circle) represents the standard deviation, and the asterisk indicates the range.

**Figure 5.**
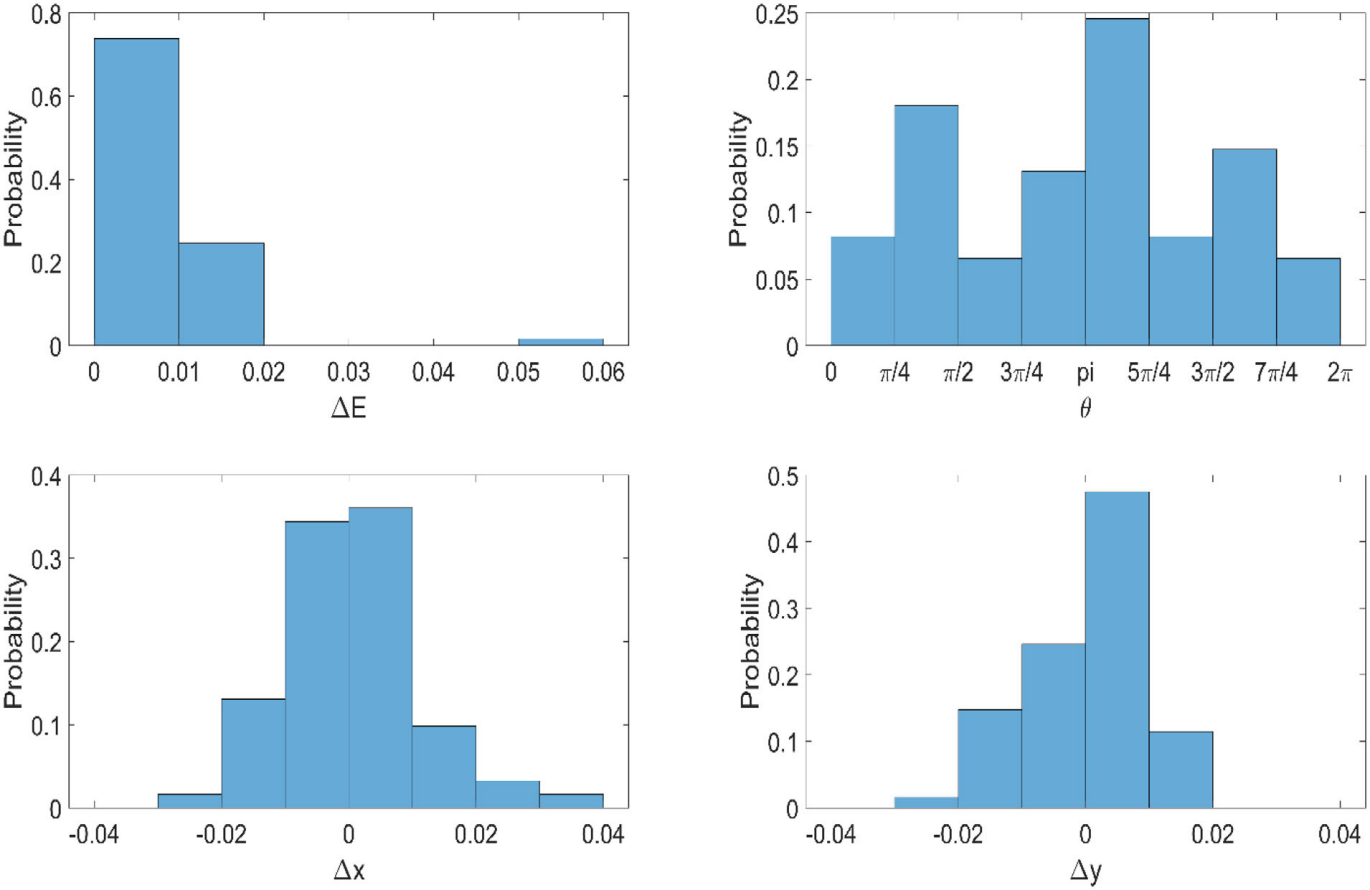
Histograms of the within-subject differences in subjective white points. Color difference (Δ*E*) and direction (θ) (top) and change in *x*, Δ*x*, *y*, and Δ*y* (bottom). Here, the vector Δ*E* is given by $\Delta E=\sqrt{{\left(u_{test}^{{}^{'}}-u_{control}^{{}^{'}}\right)}^{2}+{\left(v_{test}^{{}^{'}}-v_{control}^{{}^{'}}\right)}^{2}}$, and the angle of the vector (θ) is given by $\theta =\tan^{-1}\left(\left(v_{test}^{{}^{'}}-v_{control}^{{}^{'}}\right)/\left(u_{test}^{{}^{'}}-u_{control}^{{}^{'}}\right)\right)$.

For this within-subject comparison, the chromaticity coordinates of eyes with the SW-filtering test contact lens (*n* = 58; *x* = 0.345, *y* = 0.325, *u*′ = 0.222, *v*′ = 0.470) was not significantly different from eyes with the clear contact lens (*n* = 58; *x* = 0.344, *y* = 0.325, *u*′ = 0.223, *v*′ = 0.471) (see Figure 4, Table 1). This was also true when the subjects were separated by age into young (18–39 years, *n* = 31) and older (40–65 years, *n* = 27) groups. Within-subject differences indicate that the vast majority of subjects had a color difference (Δ*E*) less than 0.01 with no systematic bias in the color direction (θ). Similarly, within-subject differences in *x*, Δ*x*, *y*, and Δ*y* were consistently less than 0.02 (Figure 5). We also measured the energy required to make the matches (illuminance) and found no differences across lens types or age (Table 2). Although participants were not directly asked about their subjective experiences wearing the lens, no participants indicated noticing a difference that specifically related to the SW-filtering test lens.

**Table 1.** Chromaticity for the younger (left), older (middle), and combined (right) groups with SW-filtering test and clear control lenses in the per-protocol (cohort) population. Min = minimum; Max = maximum.

	Age group: 18–39 years		Age group: 40–65 years		Total	
	Test lens (*n* = 31)	Control lens (*n* = 31)	Test lens (*n* = 27)	Control lens (*n* = 27)	Test lens (*n* = 58)	Control lens (*n* = 58)
Color appearance (*u*′)						
Mean (SD)	0.222 (0.004)	0.223 (0.005)	0.223 (0.007)	0.223 (0.005)	0.222 (0.005)	0.223 (0.005)
Median	0.222	0.222	0.223	0.223	0.222	0.222
Min–Max	0.212–0.233	0.212–0.236	0.210–0.240	0.213–0.232	0.210–0.240	0.212–0.236
Color appearance (*v*′)						
Mean (SD)	0.471 (0.007)	0.471 (0.007)	0.468 (0.015)	0.470 (0.007)	0.470 (0.011)	0.471 (0.007)
Median	0.472	0.470	0.470	0.470	0.471	0.470
Min–Max	0.459–0.486	0.459–0.485	0.400–0.486	0.457–0.486	0.400–0.486	0.457–0.486
Color appearance (*x*)						
Mean (SD)	0.345 (0.010)	0.344 (0.010)	0.345 (0.012)	0.345 (0.011)	0.345 (0.011)	0.344 (0.011)
Median	0.343	0.344	0.344	0.344	0.343	0.344
Min–Max	0.332–0.368	0.322–0.371	0.317–0.366	0.325–0.370	0.317–0.368	0.322–0.371
Color appearance (*y*)						
Mean (SD)	0.326 (0.010)	0.325 (0.010)	0.324 (0.011)	0.325 (0.012)	0.325 (0.010)	0.325 (0.011)
Median	0.326	0.325	0.324	0.323	0.325	0.325
Min–Max	0.308–0.349	0.308–0.348	0.308–0.353	0.304–0.346	0.308–0.353	0.304–0.348

**Table 2.** Illuminance for the younger (left), older (middle), and combined (right) groups with SW-filtering test and clear control lenses in the per-protocol (cohort) population.

	Illuminance (lux)					
	Age group: 18–39 years		Age group: 40–65 years		Total	
	Test lens (*n* = 31)	Control lens (*n* = 31)	Test lens (*n* = 27)	Control lens (*n* = 27)	Test lens (*n* = 58)	Control lens (*n* = 58)
Mean (SD)	113 (6)	113 (6)	116 (5)	116 (6)	114 (5)	114 (6)
Median	115	113	115	117	115	115
Min–Max	103–125	102–123	104–126	103–128	104–126	102–128

### Modeling study: Results of the analysis of hyperspectral images

**Figure 6.**
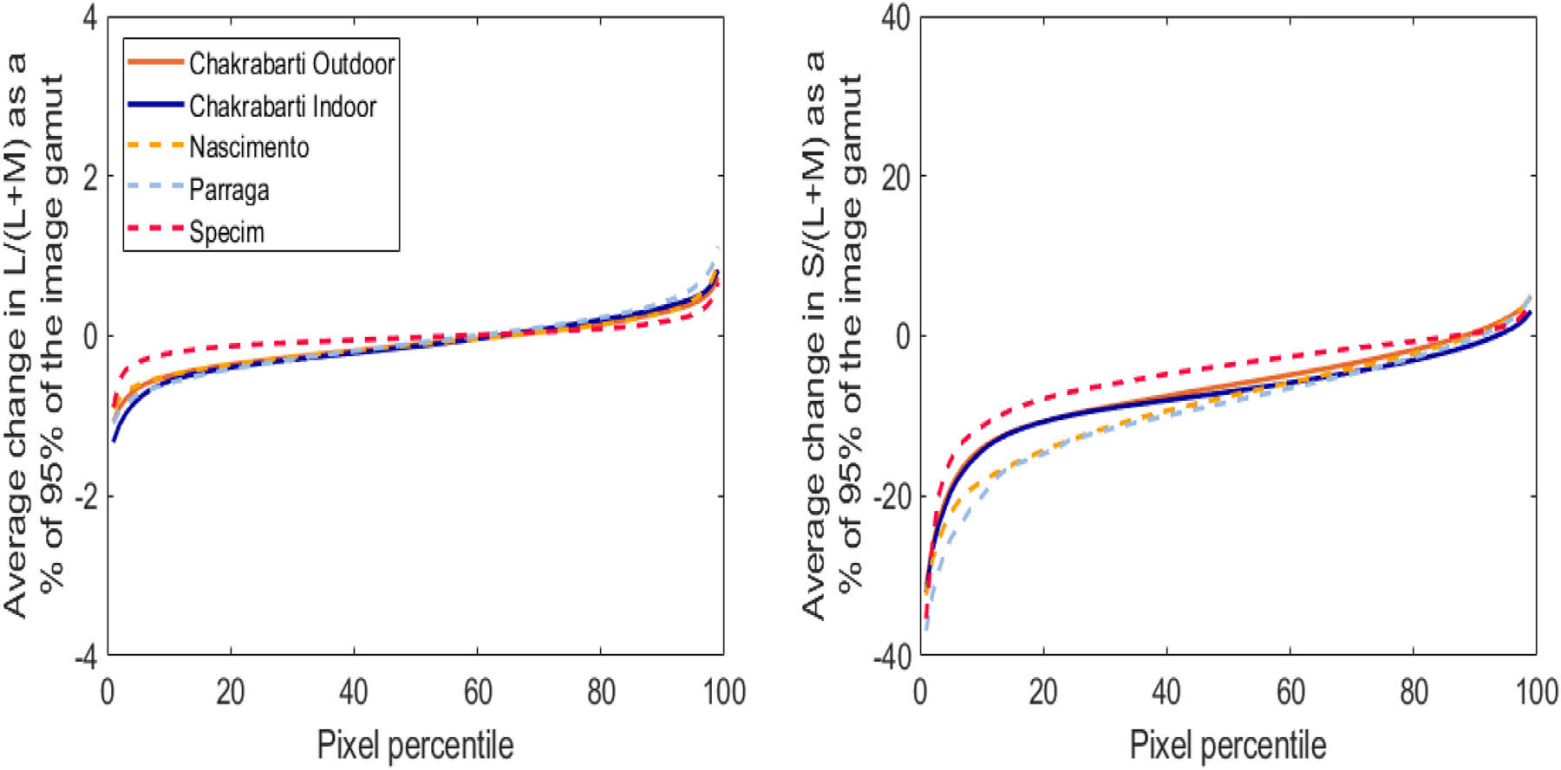
Percentile changes in chromatic contrast for L/(L+M) (left) and S/(L+M) (right) as a percentage of the original image gamut for age = 20 years and macular pigment optical density = 0.35. The collective dataset is comprised of five sets of hyperspectral images: Four image sets were obtained from the literature: Chakrabarti and Zickler (2011); outdoor, Chakrabarti & Zickler (2011); indoor, Nascimento et al. (2002); and Parraga et al. (1998). One image set was acquired using the Specim IQ hyperspectral camera.

Calculated chromatic contrast changes in L/(L+M) as a percentage of 95% of the unfiltered image gamut for each dataset show that the test lens is predicted to cause very little change in L/(L+M) chromatic contrast. Results vary by scene, and each dataset is composed of different scenes, so we present all results and report the range across datasets herein. L/(L+M) chromatic contrast is predicted to change by, at most, –1.4% and +1.1%. Between 1% and 16% of pixels (for different datasets) exhibit L/(L+M) chromatic contrast reductions of more than 0.5%, and between 1% and 7% of pixels exhibit L/(L+M) chromatic contrast enhancements of more than 0.5%. Although the change is minor, between 35% and 67% of pixels exhibit L/(L+M) chromatic contrast reductions, and the average reduction is between 0.0% and 0.1% (Figure 6, left).

Conversely, calculated chromatic contrast changes in S/(L+M) as a percentage of 95% of the unfiltered image gamut show that the test lens is predicted to generally degrade S/(L+M) chromatic contrast. Results varied slightly by scene and dataset such that the S/(L+M) chromatic contrast for individual pixels was predicted to change by, at most, –36.9% and +5.0%. Between 82% and 92% of pixels exhibited S/(L+M) chromatic contrast reductions of more than 0.5%, and between 5% and 10% of pixels exhibited S/(L+M) chromatic contrast enhancements of more than 0.5%. Between 87% and 94% of pixels exhibited S/(L+M) chromatic contrast reductions, and the average reduction was between 3.6 and 8.2% (Figure 6, right).

**Figure 7.**
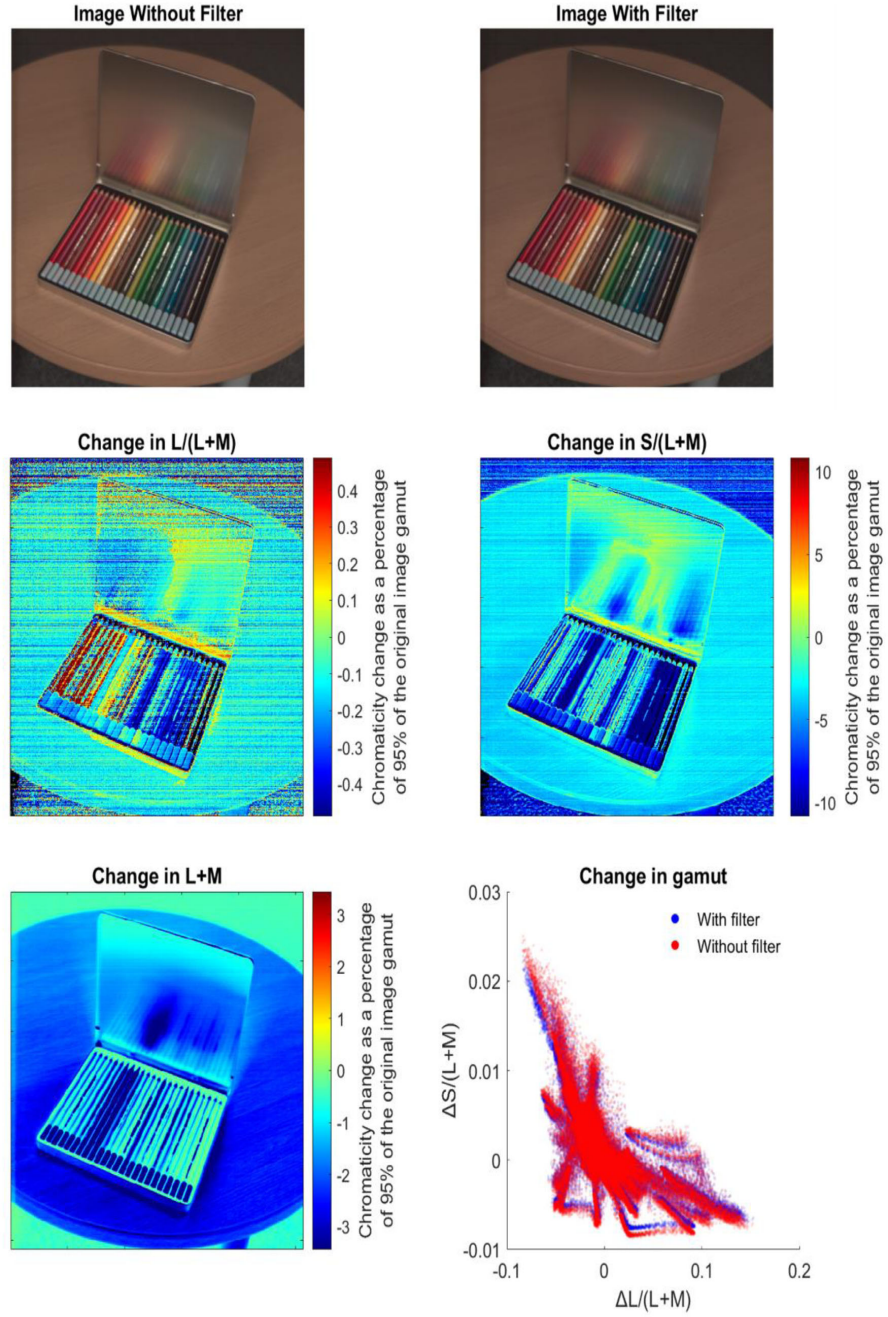
Hyperspectral image of a colored pencil set and assessments of the impact of the filter. (Top left) Original hyperspectral image recast in RGB. (Top right) Filtered hyperspectral image recast in RGB. (Middle left) Change in L/(L+M) as a percentage of 95% of the original image gamut. (Middle right) Change in S/(L+M) as a percentage of 95% of the original image gamut. (Bottom left) Change in L+M as a percentage of 95% of the original image gamut. (Bottom right) Scatterplot of the chromatic contrast of pixels in the image shown with (blue) and without (red) the filter.

A single example of a hyperspectral image of a color pencil set (from the custom Specim IQ dataset) is provided to illustrate the impact of the filter (Figure 7). Qualitatively, comparing the image with and without the filter (Figure 7, top row), the image appears a bit darker, but the colors are generally well represented in the filtered image. Quantitatively, we see that along L/(L+M) some of the red colors are enhanced slightly by as much as 0.5% and some of the blue colors are degraded slightly by as much as 0.5%. Along S(L+M), most of the image content is degraded, and the orange and blue were most degraded by as much as 10% (Figure 7, middle row). The luminance of the image was reduced slightly by as much as 3% (Figure 7, bottom left). The gamut is generally well preserved with some contraction along S/(L+M), again driven by the orange and blue surfaces in the image (Figure 7, bottom right).

## Discussion

The term “color” is used in many different ways across numerous fields, but, broadly, color is a perception. Unlike many perceptions, however, the perception of color can be very precisely quantified using the CIE colorimetry system. We used a tristimulus colorimeter along with a spectral radiometer and spectral computations based on color matching functions. Subjects were asked to mix three primaries to achieve a perfect white (a color sensation without hue). This was done on subjects wearing a SW-filtering test contact lens in one eye and a clear contact lens in the other. Based on this contralateral design of the clinical phase of our study, with 58 subjects younger and older, we found that subtle tinting of a contact lens in the violet/blue region of the spectrum did not alter color appearance values in normal trichromatic subjects. Note that we tested color vision monocularly using a contralateral design. Hence, it is possible that wearing the lens in both eyes using a between-subject design may have yielded different results. This, however, seems unlikely given the fact that color perception tends to be yoked (e.g., Roth, Pelizzone, & Hermès, 1989), nor is it uncommon to find intraocular differences in natural chromophores (e.g., yellowing of the crystalline lens) (Artigas et al., 2012) that exceeds the tinting in the test lens.

**Figure 8.**
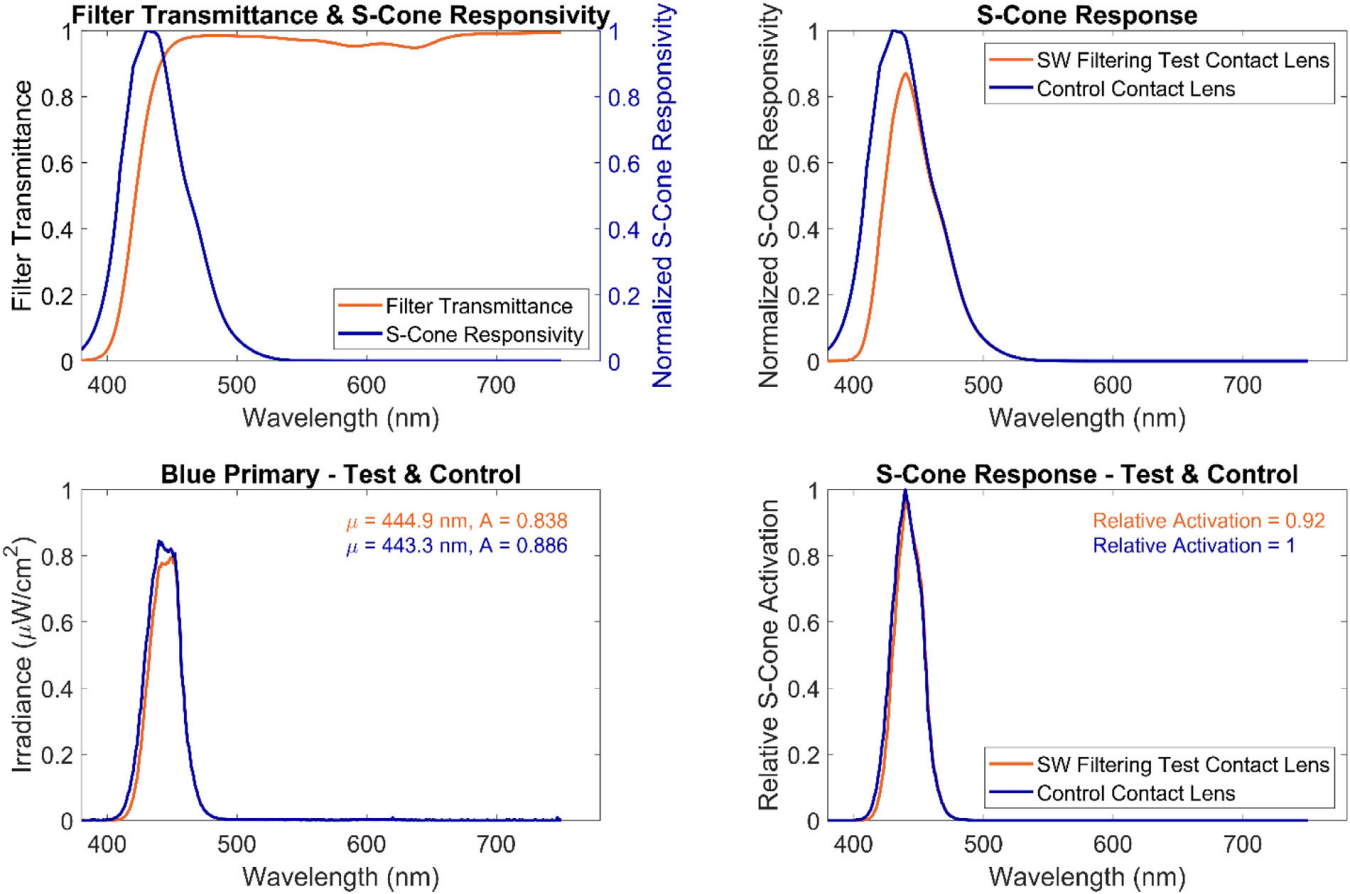
(Upper left) Filter transmittance (orange) and the normalized S-cone responsivity with no lens (blue). (Upper right) Normalized S-cone responsivity after applying the transmittance functions of either the SW-filtering test (orange) or the clear control (blue) contact lens. (Bottom left) Spectral radiance of the blue primary filtered by the SW-filtering test (orange) and the clear control (right) contact lenses. The peak amplitude and wavelength of the best-fit Gaussian is shown at the top right. (Bottom right) Relative S-cone activation to the blue primary with the SW-filtering test (orange) and the clear control (right) contact lenses. The cumulative total relative S-cone activation is shown in the top right.

Tinting contact lenses has been suggested as a means of influencing chromatic discrimination (not color appearance) in patients with color vision deficiencies (Elsherif, Salih, Yetisen, & Butt, 2021). Heavy tinting (such as is sometimes done with lenses used in sports) has been shown to influence chromatic discrimination in normal subjects (Harris & Cabrera, 1976; Laxer, 1990). The effects of tinting as one might see in more common use on color appearance, however, has not been tested until this study. Our results suggest that the effects of a modest SW filter on the white point are negligible. However, the SW filter did reduce S-cone responsivity by more than 20% for wavelengths below 433 nm, and the blue primary of the colorimeter has a full-width-at-half-maximum bandwidth spanning the range of 428 to 458 nm. As a result, the SW filter we tested in this study had a very modest impact on the blue primary of the tristimulus colorimeter, shifting the peak wavelength by only 1.6 nm and decreasing the amplitude of the irradiance by only 5.4%, and reducing the relative S-cone response by 8% (Figure 8). Given the modest impact of the SW filter on the blue primary, it is not incredibly surprising that effects on color perception were negligible. Whereas this result may not be terribly surprising, it is important to note that this type of primary is generally similar to those found in common LED display technology, where the blue primary of the display does not emit much light below 428 nm. Thus, this null result suggests that the SW-filtering lens does not adversely impact color perception of displays with primaries that are similar to the colorimeter presented herein.

Moreover, any functional impact of tinted lenses will be realized in the natural world through their effect on light in natural scenes. To estimate the impact of a SW-filtering lens on color perception in natural environments, we modeled the visual responses of individual observers to light in the real world. Summary statistics of the predicted effects of the tested tinted lenses on the gamut of colors available to a standard observer in a series of hyperspectral images acquired in natural scenes indicate that the SW-filtering lenses are expected to degrade L/(L+M) chromatic contrast by 0% to 0.1% on average and to degrade S/(L+M) chromatic contrast by between 3.6% and 8.2% on average. In summary, the SW-filtering lenses are generally not expected to change the gamut of color contrasts appreciably; however, substantial degradation of S/(L+M) contrast is expected in a minority of scene content.

Evidence in the literature supports the notion that visual adaptation occurs over multiple time scales (Wark, Fairhall, & Rieke, 2009). Short-term changes in perceived contrast have been observed with chromatic contrast adaptation for a duration of only 1 hour (Tregillus & Webster, 2014). However, our experiments were conducted after each participant wore the contact lenses for about 25 minutes. The total experimental duration was approximately 2 hours, and the experiments was not designed to assess adaptation. It is thus plausible that adaptation was partially responsible for the consistency in white point observed comparing clear and SW-filtering contact lenses.

## Conclusion

“I wept when I saw the color of the sea—how can a mere color make one cry?” (Ludwig Boltzmann, as quoted Greenstein, 1991). Color perception is profoundly important. Eyecare professionals are at once both willing to embrace tinted lenses to improve quality of life while being concerned that such lenses may degrade color perception. In this study, the impact of a short-wave filter on color appearance was assessed experimentally by tricolorimetry and theoretically by simulating a standard observer in a series of hyperspectral images acquired in natural scenes. Results indicate that the short-wave filtering lens that was evaluated generally does not appreciably alter color appearance as predicted by the model.
